# Data on metabolic profiling of spongy tissue disorder in *Mangifera indica* cv. Alphonso

**DOI:** 10.1016/j.dib.2018.11.140

**Published:** 2018-12-04

**Authors:** Pranjali S. Oak, Ashish B. Deshpande, Keshav H. Pujari, Shrikant S. Prabhudesai, Ashok P. Giri, Vidya S. Gupta

**Affiliations:** a Plant Molecular Biology Unit, Division of Biochemical Sciences, CSIR-National Chemical Laboratory, Pune 411008, India; b Dr. Balasaheb Sawant Konkan Agriculture University, Dapoli 415712, India; c Academy of Scientific and Innovative Research (AcSIR), Ghaziabad 201002, India; d Indian Institute of Science Education and Research, Pune 411008, India

**Keywords:** Volatiles, Fatty acid composition, Spongy tissue disorder

## Abstract

Data in this article presents aroma volatiles and fatty acids composition of mesocarp specific malady namely spongy tissue disorder in *Mangifera indica* cv. Alphonso. Quantitative changes in various aroma volatile compound classes as well as saturated and unsaturated fatty acids in spongy tissue vis-à-vis healthy mesocarp have been analyzed throughout the development of the disorder. Statistical data analysis correlates the dynamic changes in the aroma volatiles composition to that of the modulation in the fatty acids profile.

**Specification table**

**Table:** 

Subject area	*Biology, chemistry*
More specific subject area	*Aroma volatile and fatty acid composition of healthy, spongy affected and spongy control Alphonso mango fruit*
Type of data	*Table, graph, figure*
How data were acquired	*GC-MS: 7890B GC system Agilent Technologies coupled with Agilent 5977A MSD (Agilent technologies*^*®*^*, CA, U.S.A.)*
Data format	*Analyzed*
Experimental factors	*Alphonso mango fruits were harvested at mature raw stage from the trees and ripened in hay boxes. At various ripening stages, healthy and spongy tissue mesocarp of fruits were recovered. Pulp/mesocarp from healthy fruits, spongy tissue as well as healthy part around spongy tissue from affected fruits were collected, snap frozen in liquid nitrogen and stored at −80* *°C.*
Experimental features	*Aroma volatiles were extracted by solvent extraction method in dichloromethane and acetone (80:20), Fatty acids were methylated by transesterification to generate Fatty acid methyl esters (FAMEs) which were then extracted using chloroform. Metabolite identification and quantification was done by GC-MS-FID. Activity of cell wall degrading enzymes was determined using specific biochemical assay for individual enzyme. Relative transcript abundance of gene was determined using quantitative reverse transcriptase polymerase chain reaction.*
Data source location	*Plant Molecular Biology Unit, Division of Biochemical Sciences, CSIR-National Chemical Laboratory, Pune 411 008, (M. S.) India.*
Data accessibility	*Data provided within this article*

**Value of the data**

• Investigated data highlight the metabolic changes occurring in the spongy tissue disorder of Alphonso mango, which is valuable to researchers working on diseases or disorders of fruits.
• Ripening related data have shown decreased enzymatic activity in mesocarp from the spongy area as compared to that in the healthy fruits. This suggests the arrest of ripening process of mesocarp which in turns affects the internal fruit morphology without affecting external physiology of fruit.
• Reduced levels of lactones and furanones; the key volatiles of Alphonso mango flavor highlight the poor quality of spongy tissue affected Alphonso mango, which is of value to domestic and export market chain.
• Analyzed data highlight the significantly elevated levels of terpenes, green leafy volatiles and linoleic acid content with exclusive presence of oxygenated terpenes in the spongy tissue compared to the healthy and the spongy control fruits throughout the ripening stages.
• Higher ratio of linoleic acid to linolenic acid in spongy mesocarp suggests the probable lipid peroxidation in affected tissue.
• Present metabolic and enzymatic data are a foundation work for the development of on line non destructive sensor technology or visualization technique, to segregate the spongy tissue disordered fruits from that of healthy, which can ultimately be beneficial to mango farmers and exporters.

## 1 Data

**Fig. 1:**
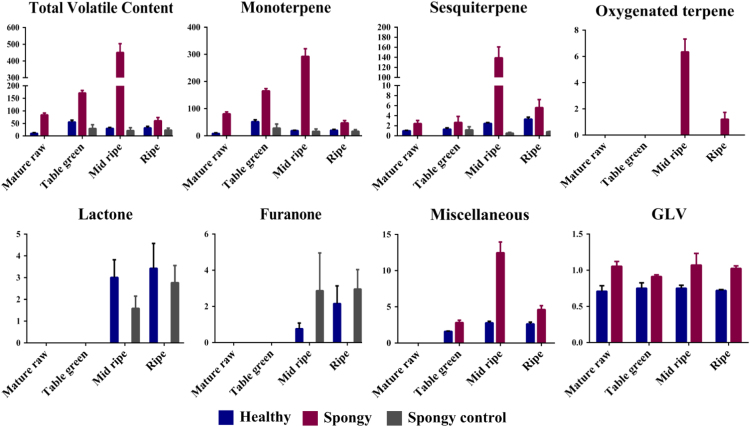
Total volatile concentration – Average concentration (µg/g of tissue) of compound classes identified in various ripening stages of Healthy, Spongy and Spongy control tissues of Alphonso mango. Vertical bars show the standard error calculated from the five biological replicates. *X*-axis represents the ripening stages of Alphonso mango while *Y*-axis denotes the concentration of compounds (µg/g of tissue).

**Fig. 2:**
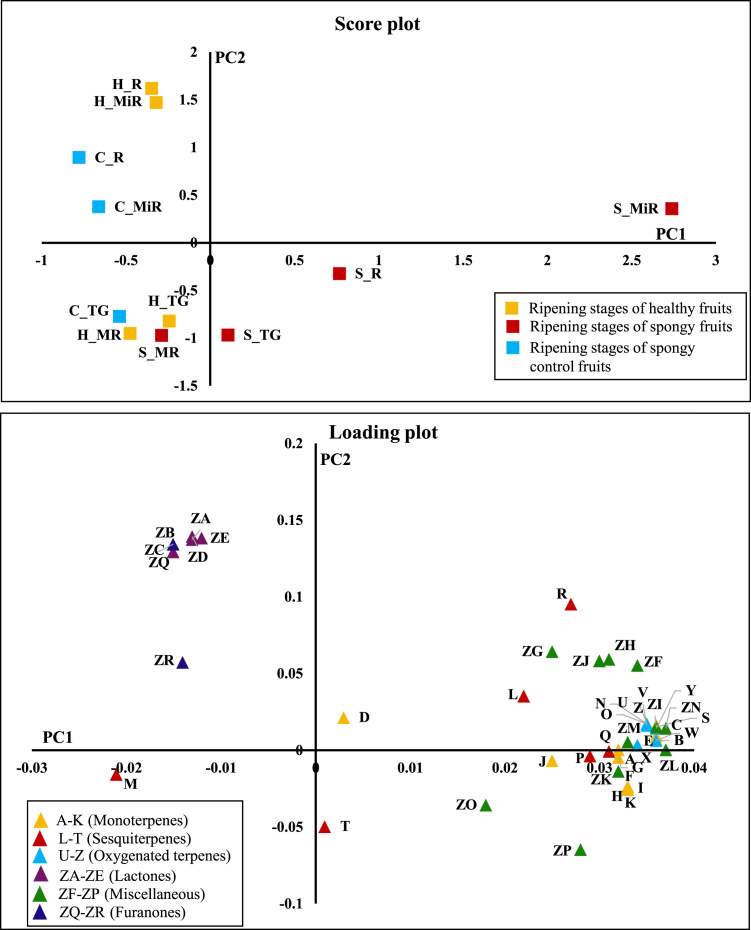
Principle Component Analysis of volatiles – 45 individual volatile compounds contributed by the 4 different ripening stages (Mature raw: MR, Table green: TG, Mid ripe: MiR, Ripe: R) of Healthy (H), Spongy (S) and Spongy control (C) tissues. Average concentration value of each volatile compound from five biological replicates has been considered.

**Fig. 3:**
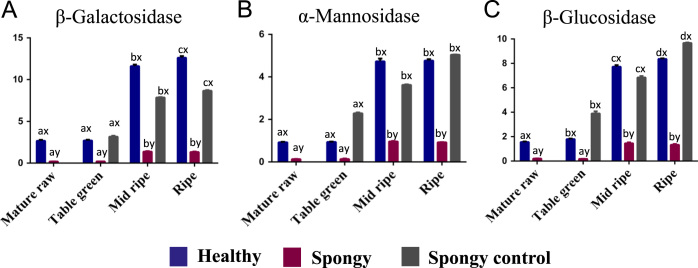
Ripening enzyme analysis – Enzyme activity of three different enzymes, (A) β-Galactosidase, (B) α-Mannosidase and (C) β-Glucosidase at 4 different ripening stages in healthy, spongy and spongy control tissues of Alphonso mango fruit. Vertical bars represent the standard error calculated from the five biological replicates. Difference between the stages for each tissue set was significant (*p* ≤ 0.05) for each enzyme if the alphabets (a, b, c….) are different. Difference between the tissue sets at each stage was significant (*p* ≤ 0.05) for each enzyme if the alphabets (x, y) are different. *X*-axis denotes the ripening stages of the Alphonso fruit while *Y*-axis denotes concentration of the enzyme in terms of amount of pNp released (mM/g of tissue).

Total 45 different aroma volatiles have been identified from mesocarp of three different tissue sets (Healthy, Spongy and Spongy control) of Alphonso mango fruit at four stages of fruit ripening. These aroma volatiles belong to different compound classes such as Monoterpenes (11), Sesquiterpenes (09), Lactones (05), Oxygenated terpenes (06), Furanones (02) and miscellaneous compounds (12), which includes alkanes, alkenes, alcohols, esters, green leafy volatiles etc. (Table 1, Figs. 1 and 2). Pandit et al. [1] have analyzed the stage specific dynamics of aroma volatile compounds of *Mangifera indica* cv. Alphonso pulp. Dominance of terpenes was seen at the early stages of ripening i.e. in unripe fruits whereas lactones and furanones were considered to be the ripening markers because of their exclusive appearance at the later stages of ripening. In case of the spongy tissue, significantly higher concentration of terpenes in mid ripe and ripe stages and loss of lactones and furanones in these stages suggested the slower synthesis of specific marker metabolites of ripening in spongy tissue. Further decreased activity of three major ripening related enzymes (β-D-Galactosidase, α-D-Mannosidase and β-D-Glucosidase) (Fig. 3) lead to the accumulation of respective substrates. These observations suggest arrest of ripening of Alphonso fruit in the spongy tissue, which in turn affects the physicochemical properties of the mesocarp. In addition to terpenes, significant increase in green leafy volatiles concentration was observed in case of the spongy tissue as compared to that in the healthy and the spongy control fruits in all the stages of ripening. Terpenes and green leafy volatiles are important defense compounds of the plants. These compounds are released during fruit development, herbivores attack or tissue damage [2]. Hence, increased concentration of terpenes and green leafy volatiles in the spongy tissue suggests the stress condition inside the fruit. This has also been confirmed by the exclusive presence of 2,4-decadienal in the spongy tissue, which is a primary oxidation product of Linoleic acid.

**Table 1:** Volatile composition of Alphonso mango in pulp.

**Compound**	**Healthy**				**Spongy**				**Spongy control**		
	Mature Raw	Table green	Mid Ripe	Ripe	Mature Raw	Table green	Mid Ripe	Ripe	Table green	Mid ripe	Ripe
**Monoterpenes**											
α-Pyronene	n.d.^ax^	n.d.^ax^	n.d.^ax^	n.d.^ax^	n.d.^ax^	n.d.^ax^	0.87^ay^	0.90^ay^	n.d.^ax^	n.d.^ax^	n.d.^ax^
α-Pinene	n.d.^ax^	n.d.^ax^	n.d.^ax^	n.d.^ax^	n.d.^ay^	n.d.^ay^	0.61^by^	0.36^cy^	n.d.^ax^	n.d.^ax^	n.d.^ax^
camphene	n.d.^ax^	n.d.^ax^	n.d.^ax^	n.d.^ax^	n.d.^ay^	n.d.^ay^	0.11^by^	0.01^cy^	n.d.^ax^	n.d.^ax^	n.d.^ax^
(-)-β-Pinene	n.d.^ax^	0.02^ax^	0.14^ax^	0.30^ax^	n.d.^ax^	0.48^by^	0.14^ax^	0.07^ax^	0.18^ax^	0.15^ax^	0.04^ax^
β-Myrcene	0.32^ax^	0.15^ax^	0.24^ax^	0.21^ax^	0.16^ax^	0.81^ax^	5.49^by^	1.07^ax^	0.30^ax^	0.22^ax^	0.14b^ax^
d-Limonene	0.05^ax^	0.17^ax^	0.25^ax^	0.10^ax^	0.26^ax^	0.64^by^	1.05^cy^	0.17^dx^	0.24^ax^	0.19^bx^	0.04^bx^
β-Pyronene	0.16^ax^	0.09^ax^	0.08^ax^	0.13^ax^	0.11^ax^	0.15^ax^	0.37^by^	0.09^ax^	n.d.^az^	n.d.^az^	n.d.^az^
(Z)-Ocimene	5.26^ax^	34.35^bx^	12.14^ax^	13.15^ax^	52.97^ay^	108.91^by^	186.61^cy^	28.70^dy^	24.22^ax^	13.84^bx^	14.32^bx^
(E)-Ocimene	0.46^ax^	2.32^bx^	1.01^ax^	1.03^ax^	3.67^ay^	6.99^by^	12.30^cy^	1.97^dx^	1.24^ax^	0.66^bx^	0.63^bx^
α-Naginatene	n.d.^ax^	n.d.^ax^	n.d.^ax^	n.d.^ax^	n.d.^ax^	n.d.^ax^	0.11^ax^	0.21^ay^	n.d.^ax^	n.d.^ax^	n.d.^ax^
Neo-allo-ocimene	2.17^ax^	14.90^bx^	5.19^ax^	5.56^ax^	23.56^ay^	47.08^ay^	84.81^by^	13.48^cx^	2.42^ax^	1.50^ax^	1.50^ax^
**Sesquiterpenes**											
Copaene	0.02^ax^	0.78^bx^	1.10^cx^	1.13^dx^	0.82^ay^	0.94^by^	1.20^cy^	1.11^dy^	0.05^ax^	0.02^ax^	0.02^ax^
d-longifolene	0.65^ax^	0.04^bx^	0.19^bx^	0.24^bx^	0.19^ay^	0.22^bx^	n.d.^cx^	n.d.^cy^	0.47^ax^	0.11^ax^	0.52^ax^
Caryophyllene	0.20^ax^	0.32^bx^	0.28^bx^	0.76^bx^	0.91^ay^	0.92^ay^	86.22^bx^	2.27^by^	0.41^ax^	0.26^ax^	0.24^ax^
Humulene	0.14^ax^	0.17^ax^	0.22^ax^	0.51^ax^	0.51^ax^	0.54^ax^	49.42^by^	1.28^ay^	0.24^ax^	0.14^ax^	0.02^ax^
α-Guaiene	n.d.^ax^	n.d.^ax^	n.d.^ax^	0.66^ax^	n.d.^ax^	n.d.^ax^	0.19^ay^	0.27^ay^	n.d.^ax^	n.d.^ax^	n.d.^ax^
α-Selinene	n.d.^ax^	n.d.^ax^	n.d.^ax^	n.d.^ax^	n.d.^ax^	n.d.^ax^	0.17^ay^	0.19^ay^	n.d.^ax^	n.d.^ax^	n.d.^ax^
Farnesan	n.d.^ax^	n.d.^ax^	0.67^bx^	n.d.^bx^	n.d.^ay^	n.d.^ay^	1.03^by^	0.34^cy^	n.d.^ax^	n.d.^ax^	n.d.^ax^
δ-Cadinene	n.d.^ax^	n.d.^ax^	n.d.^ax^	n.d.^ax^	n.d.^ax^	n.d.^ax^	0.87^by^	0.14^cx^	n.d.^ax^	n.d.^ax^	n.d.^ax^
Longicyclene	n.d.^ax^	n.d.^ax^	n.d.^ax^	n.d.^ax^	n.d.^ax^	0.02^by^	n.d.^ax^	n.d.^ax^	0.03^ax^	0.08^ax^	0.06^ax^
**Lactones**											
γ-Butyrolactone	n.d.^ax^	n.d.^ax^	0.96^bx^	0.68^bx^	n.d.^ax^	n.d.^ax^	n.d.^ay^	n.d.^ay^	n.d.^ax^	0.28^bx^	0.65^bx^
γ-Hexalactone	n.d.^ax^	n.d.^ax^	0.34^bx^	0.58^bx^	n.d.^ax^	n.d.^ax^	n.d.^ay^	n.d.^ay^	n.d.^ax^	0.36^bx^	0.44^bx^
δ-Hexalactone	n.d.^ax^	n.d.^ax^	0.47^bx^	0.47^bx^	n.d.^ax^	n.d.^ax^	n.d.^ay^	n.d.^ay^	n.d.^ax^	0.31^bx^	0.69^bx^
γ-Octalactone	n.d.^ax^	n.d.^ax^	0.96^bx^	1.34^bx^	n.d.^ax^	n.d.^ax^	n.d.^ay^	n.d.^ay^	n.d.^ax^	0.53^bx^	0.81^bx^
δ-Octalactone	n.d.^ax^	n.d.^ax^	0.28^bx^	0.36^bx^	n.d.^ax^	n.d.^ax^	n.d.^ay^	n.d.^ay^	n.d.^ax^	0.08^bx^	0.17^bx^
**Oxygenated terpenes**											
2,6-Dimethyl-3(E),5(E),7-octatriene-2-ol	n.d.^ax^	n.d.^ax^	n.d.^ax^	n.d.^ax^	n.d.^ax^	n.d.^ax^	1.50^by^	0.10^cy^	n.d.^ax^	n.d.^ax^	n.d.^ax^
Cis-Verbenol	n.d.^ax^	n.d.^ax^	n.d.^ax^	n.d.^ax^	n.d.^ax^	n.d.^ax^	2.70^by^	0.31^cy^	n.d.^ax^	n.d.^ax^	n.d.^ax^
Carveol	n.d.^ax^	n.d.^ax^	n.d.^ax^	n.d.^ax^	n.d.^ax^	n.d.^ax^	0.16^by^	0.10^cy^	n.d.^ax^	n.d.^ax^	n.d.^ax^
Nerolidol	n.d.^ax^	n.d.^ax^	n.d.^ax^	n.d.^ax^	n.d.^ax^	n.d.^ax^	0.63^by^	0.53^cy^	n.d.^ax^	n.d.^ax^	n.d.^ax^
Caryophyllene oxide	n.d.^ax^	n.d.^ax^	n.d.^ax^	n.d.^ax^	n.d.^ax^	n.d.^ax^	0.50^by^	0.10^cy^	n.d.^ax^	n.d.^ax^	n.d.^ax^
Humulene oxide	n.d.^ax^	n.d.^ax^	n.d.^ax^	n.d.^ax^	n.d.^ax^	n.d.^ax^	0.86^by^	0.07^cy^	n.d.^ax^	n.d.^ax^	n.d.^ax^
**Furanones**											
Mesifuran	n.d.^ax^	n.d.^ax^	0.33^bx^	2.06^bx^	n.d.^ax^	n.d.^ax^	n.d.^ax^	n.d.^ax^	0.08^ax^	1.526^bx^	1.69^bx^
Ethyl maltol	n.d.^ax^	n.d.^ax^	0.43^bx^	0.10^bx^	n.d.^ax^	n.d.^ax^	n.d.^ax^	n.d.^ax^	0.06^ax^	1.34^bx^	1.25^bx^
**Miscellaneous**											
Decane	n.d.^ax^	n.d.^ax^	0.19^bx^	0.16^bx^	n.d.^ax^	n.d.^ax^	0.67^by^	0.16^cx^	n.d.^ax^	n.d.^ax^	Trace^b^
Dodecane	n.d.^ax^	n.d.^bx^	0.20^cx^	0.18^cx^	n.d.^ax^	0.17^by^	0.34^cy^	0.17^dx^	n.d.^ax^	n.d.^bx^	Trace^b^
1-dodecene	n.d.^ax^	0.35^ax^	1.39^bx^	1.39^bx^	n.d.^ax^	1.18^by^	1.63^cy^	1.42^bx^	n.d.^ax^	Trace^b^	Trace^b^
Isocyclocitral	n.d.^ax^	n.d.^ax^	n.d.^ax^	n.d.^ax^	n.d.^ax^	n.d.^ax^	1.52^by^	0.20^by^	n.d.^ax^	n.d.^ax^	n.d.^ax^
1-Hexadecanol	n.d.^ax^	n.d.^ax^	0.38^bx^	0.29^cx^	n.d.^ax^	n.d.^ax^	0.69^by^	0.75^by^	n.d.^ax^	n.d.^ax^	Trace^b^
Hexadecane	n.d.^ax^	0.69^bx^	0.18^cx^	0.13^cx^	n.d.^ax^	0.59^bx^	1.20^cy^	0.49^dy^	n.d.^ax^	Trace^b^	Trace^b^
Heptadecane	n.d.^ax^	0.08^bx^	n.d.^ax^	n.d.^ax^	n.d.^ax^	0.05^bx^	0.49^cy^	0.17^dy^	n.d.^ax^	n.d.^ax^	n.d.^ax^
Octadecane	n.d.^ax^	0.31^bx^	0.18^bx^	0.27^bx^	n.d.^ax^	0.48^bx^	0.78^by^	1.10^bx^	n.d.^ax^	Trace^b^	Trace^b^
Nonadecane	n.d.^ax^	0.18^bx^	0.30^bx^	0.22^bx^	n.d.^ax^	0.36^bx^	5.15^cy^	0.16^dx^	n.d.^ax^	Trace^b^	Trace^b^
Decadienal	n.d.^ax^	n.d.^ax^	n.d.^ax^	n.d.^ax^	0.05^ay^	0.07^ay^	0.18^ay^	0.16^by^	n.d.^ax^	n.d.^ax^	n.d.^ax^
5,5-dimethyl hexanal^a^	0.65^ax^	0.74^bx^	0.74^ax^	0.71^abx^	0.96^ay^	0.85^ax^	0.85^ay^	0.96^aby^	Trace^b^	Trace^b^	Trace^b^
1,5-dimethylhexyl acetate^a^	0.06^ax^	0.01^abx^	0.01^bx^	0.01^bx^	0.09^ax^	0.06^aby^	0.21^by^	0.06^abcy^	Trace^b^	Trace^b^	Trace^b^

Volatile composition (µg g^−1^ tissue) of healthy, spongy and spongy control pulp at various stages of fruit ripening of Alphonso mango. Values shown are average of biological replicates sampled for the study. Difference between the stages was significant (*p* ≤ 0.05) if the alphabets (a, b, c….) after the quantity of the compounds are different. Difference between the tissue set for each compound at each stage of ripening was significant (*p* ≤ 0.05) if the alphabets (x, y) after the quantity of the compounds are different. Mature raw stage of spongy and spongy control can not be differentiated hence, not shown separately in spongy control.

a Green leafy volatiles.

b Compound could only be detected in MS but could not be quantified by FID.

**Fig. 4:**
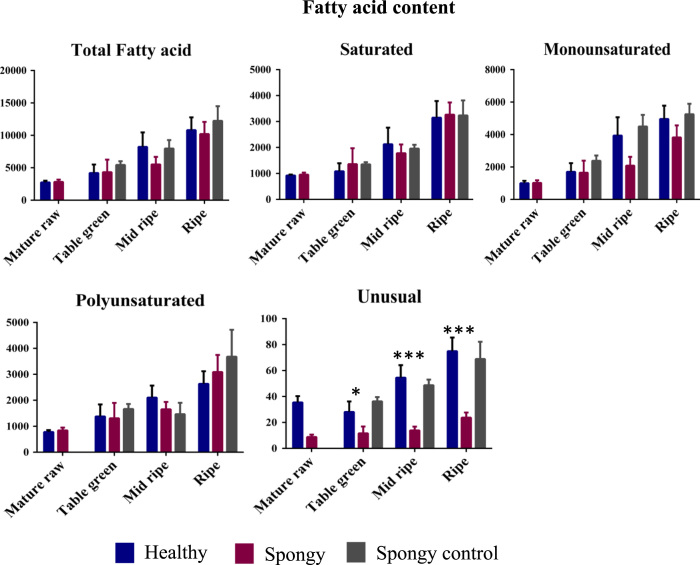
Total fatty acid concentration – Average concentration (mg/g of tissue) of fatty acid classes identified among Healthy, Spongy and Spongy control tissues of Alphonso mango fruit calculated considering five biological replicates. *X*-axis denotes stages of Alphonso fruit ripening while *Y*-axis denotes concentration of fatty acids (mg/g of tissue). (*p* ≤ 0.05= *; *p* ≤ 0.01=**; *p* ≤ 0.001=***).

**Fig. 5:**
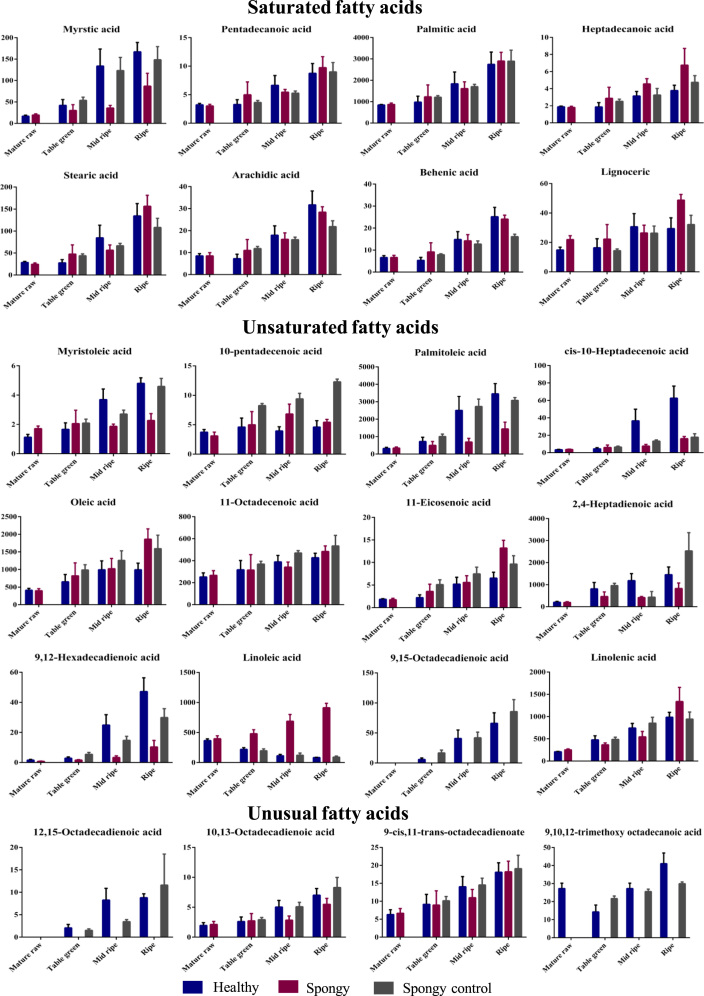
Fatty acid concentration – Average concentration of (mg/g of tissue) individual fatty acid on *Y*-axis identified at various ripening stages on *X*-axis among Healthy, Spongy and Spongy control tissues. Vertical bars represent the standard error calculated from biological replicates.

**Fig. 6:**
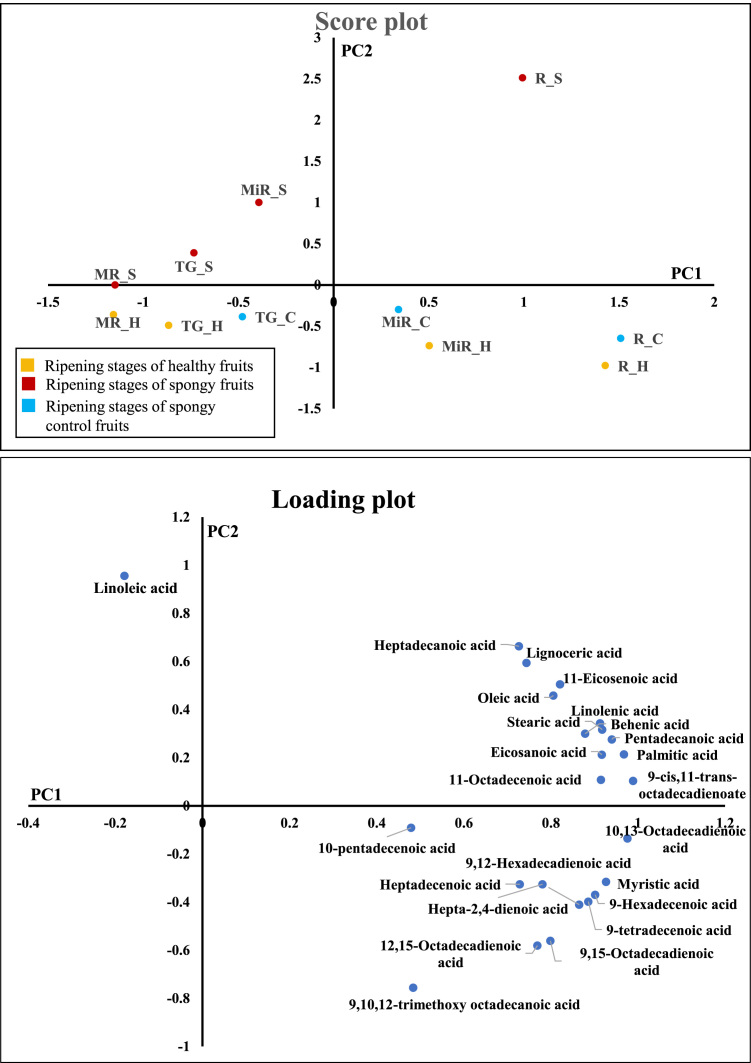
Principle Component Analysis of fatty acids – 24 individual fatty acids contributing to 4 different ripening stages (Mature Raw: MR, Table Green: TG, Mid Ripe: MiR, Ripe: R) of Healthy (H), Spongy (S) and Spongy control (C) tissue. Average concentration values of each fatty acid from five biological replicates have been considered.

**Fig. 7:**
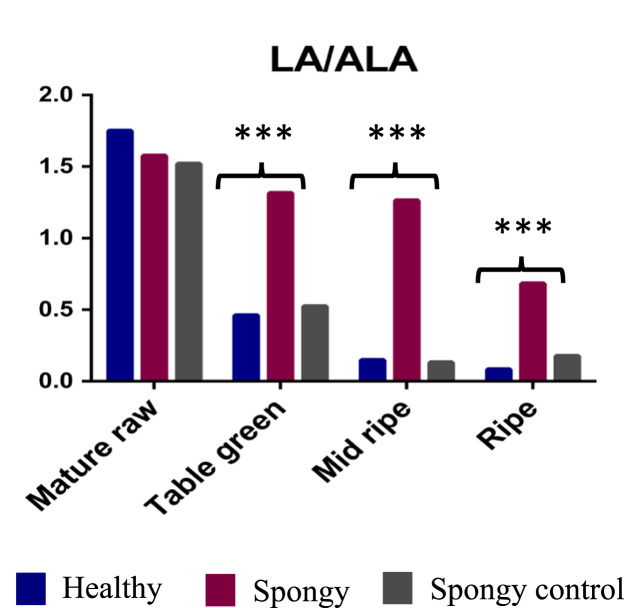
Ratio of LA/ALA concentration at various ripening stages of Alphonso mango fruit on *X*-axis among Healthy, Spongy and Spongy control tissues. (*p* ≤ 0.05= *; *p* ≤ 0.01=**; *p* ≤ 0.001=***).

Furthermore, total 24 different fatty acids were also identified (Table 2 and Fig. 4, Fig. 5, Fig. 6). Significant decrease in fatty acids such as Myristic, Myristoleic, Palmitoleic, Hepta-2,4-dienoic, cis-10-heptadecenoic, 9,12-hexadienoic acid was observed in the spongy tissue as compared to the spongy control and the healthy fruit. Similarly, significant increase in Heptadecanoic, Oleic, Linoleic, 11-Eicosenoic and Lignoceric acid was observed in the spongy tissue as compared to the spongy control and the healthy fruit. 9,15-Octadecadienoic acid, which is known as mangiferic acid, along with 12,15-Octadecadienoic acid were absent in the spongy tissue. Such absence of polyunsaturated fatty acids highlights the membrane disintegration. Enhanced process of membrane disruption and lipid peroxidation was also depicted with significant increase in the ratio of LA/ALA content (Fig. 7). Due to the higher accumulation of Linoleic acid it leads to reducing the nutritive value of fruit.

**Table 2:** Fatty acid composition of Alphonso mango in pulp.

Fatty acid	**Healthy**				**Spongy**				**Spongy control**		
	Mature raw	Table green	Mid ripe	Ripe	Mature raw	Table green	Mid ripe	Ripe	Table green	Mid ripe	Ripe
**Saturated**											
Myristic acid (C14:0)	17.00^ax^	42.25^ax^	133.96^bx^	167.04^bx^	19.51^ax^	30.18^ax^	35.91^ay^	87.04^by^	53.83^ax^	123.25^bx^	148.58^bx^
Pentadecanoic acid^a^ (C15:0)	3.22^ax^	3.29^ax^	6.63^bx^	8.74^bx^	3.01^ax^	5.00^ax^	5.44^ax^	9.74^bx^	3.65^ax^	5.27^ax^	8.99^ax^
Palmitic acid (C16:0)	844.15^ax^	982.64^ax^	1833.49^abx^	2745.87^bx^	870.49^ax^	1231.75^ax^	1616.40^ax^	2902.16^bx^	1212.53^ax^	1704.59^ax^	2893.50^bx^
Heptadecanoic acid^a^ (C17:0)	1.88^ax^	1.86^ax^	3.15^ax^	3.78^ax^	1.81^ax^	2.88^ax^	4.56^ax^	6.76^by^	2.52^ax^	3.25^ax^	4.74^ax^
Stearic acid (C18:0)	412.35^ax^	27.71^ax^	84.78^bx^	134.60^bx^	24.54^ax^	47.52^ax^	56.54^ax^	156.27^bx^	43.86^ax^	66.67^ax^	108.25^bx^
Arachidic acid (C20:0)	8.52^ax^	7.29^ax^	17.92^bx^	31.72^cx^	8.53^ax^	11.02^ax^	16.05^ax^	28.37^bx^	11.83^ax^	15.87^ax^	21.82^ax^
Behenic acid (C22:0)	6.62^ax^	5.30^ax^	14.86^bx^	25.19^cx^	6.66^ax^	9.19^abx^	14.20^bx^	24.12^cx^	7.87^ax^	12.77^bx^	16.10^bx^
Lignoceric acid (C24:0)	14.93^ax^	16.36^abx^	30.68^bx^	29.42^bx^	21.90^ax^	22.22^ax^	26.44^ax^	48.72^by^	14.36^ax^	26.27^ax^	32.18^ax^
**Mono-unsaturated**											
Myristoleic acid (C14:1, n-5)	1.13^ax^	42.25^ax^	133.96^bx^	167.04^cx^	19.51^ax^	30.18^ax^	35.91^ay^	87.04^ay^	2.09^ax^	2.71^ax^	4.59^ax^
10-pentadecenoic acid (C15:1)	3.74^ax^	4.60^ax^	3.94^ax^	4.60^ax^	3.10^ax^	5.00^abx^	6.83^bx^	5.42^abx^	8.26^ax^	9.39^ax^	12.29^ax^
Palmitoleic acid (C16:1, n-7)	321.46^ax^	722.80^ax^	2505.98^bx^	3457.97^bx^	343.13^ax^	499.48^ax^	694.76^ay^	1443.96^ay^	1011.20^ax^	2732.99^bx^	3079.49^bx^
cis-10-Heptadecenoic acid (C17:1, n-7)	3.38^ax^	4.51^ax^	36.59^bx^	62.66^cx^	3.71^ax^	6.05^ax^	7.67^ay^	16.10^ay^	6.56^ax^	13.40^ax^	17.75^ax^
Oleic acid (C18:1, n-9)	412.35^ax^	653.46^ax^	990.66^ax^	991.34^ax^	396.32^ax^	819.13^abx^	1023.18^bx^	1861.28^cy^	983.41^ax^	1259.72^bx^	1593.06^bx^
11-Octadecenoic acid (C18:1, n-7)	251.80^ax^	317.21^abx^	389.44^abx^	426.97^bx^	267.16^ax^	314.28^ax^	341.68^ax^	483.85^ax^	369.14^ax^	470.48^abx^	534.12^bx^
11-Eicosenoic acid (C20:1, n-9)	1.85^ax^	2.22^abx^	5.20^bx^	6.52^abcx^	1.76^ax^	3.59^abx^	5.58^bx^	13.20^cy^	5.11^ax^	7.47^ax^	9.64^ax^
**Poly-unsaturated**											
Hepta-2,4-dienoic acid (C7:2, n-3)	203.51^ax^	811.44^abx^	1183.37^bx^	1452.69^bcx^	194.65^ax^	464.48^abx^	694.76a^by^	1443.96^by^	960.27^ax^	436.26^ax^	2532.27^bx^
9,12-Hexadecadienoic acid (C16:2, n-4)	1.59^ax^	2.25^ax^	24.85^bx^	47.14^cx^	0.77^ax^	1.57^ax^	3.28^ay^	10.34^ay^	5.44^ax^	14.72^ax^	29.86^ax^
Linoleic acid (C18:2, n-6)	367.47^ax^	175.99^bx^	111.41^bx^	83.22^bx^	396.73^ax^	480.30^ay^	687.56^by^	913.24^cy^	195.80^ax^	120.26^ax^	89.88^ax^
9,15-Octadecadienoic acid (C18:2, n-3)	n.d.^ax^	5.92^ax^	40.85^bx^	66.15^cx^	n.d.^ax^	n.d.^ax^	n.d.^ay^	n.d.^ay^	16.87^ax^	41.95^ax^	85.64^ax^
Linolenic acid (C18:3, n-3)	209.87^ax^	381.93^ax^	744.20^bx^	985.58^bx^	251.80^ax^	365.23^ax^	543.92^ax^	1338.45^bx^	487.53^ax^	852.60^ax^	943.45^ax^
**Unusual**											
12,15-Octadecadienoic acid (C18:2)	n.d.^ax^	2.07^ax^	8.25^bx^	8.81^bx^	n.d.^ax^	n.d.^ax^	n.d.^ay^	n.d.^ay^	1.52^ax^	3.46^ax^	11.60^ax^
10,13-Octadecadienoic acid (C18:2)	1.96^ax^	2.61^ax^	5.03^bx^	7.02^bx^	2.12^ax^	2.73^ax^	2.84^ax^	5.49^bx^	2.92^ax^	5.08^ax^	8.30^ax^
9-cis,11-trans-octadecadienoic acid (C18:2)	6.30^ax^	9.15^abx^	14.04^bx^	18.09^bcx^	6.66^ax^	8.92^ax^	11.00^ax^	18.22^bx^	10.13^ax^	14.54^ax^	19.07^ax^
9,10,12-trimethoxy Octadecanoic acid (C22:3)	27.23^ax^	14.31^bx^	27.23^ax^	41.06^cx^	n.d.^ay^	n.d.^ay^	n.d.^ay^	n.d.^ay^	21.69^ax^	25.59^ax^	29.93^ax^

Fatty acid composition (µg g^−1^ tissue) of healthy, spongy and spongy control pulp at various stages of fruit ripening of Alphonso mango. Values shown are average of biological replicates sampled for the study. Difference between the stages was significant (*p* ≤ 0.05) if the alphabets (a, b, c….) after the quantity of the compounds are different. Difference between the tissue set for each compound at each stage of ripening was significant (*p* ≤ 0.05) if the alphabets (x, y…) after the quantity of the compounds are different. Mature raw stage of spongy and spongy control can not be differentiated hence, not shown separately in spongy control.

a Odd chain saturated fatty acids identified by matching mass spectrum from NIST2011 and Wiley 10^th^ edition mass spectral libraries.

**Fig. 8:**
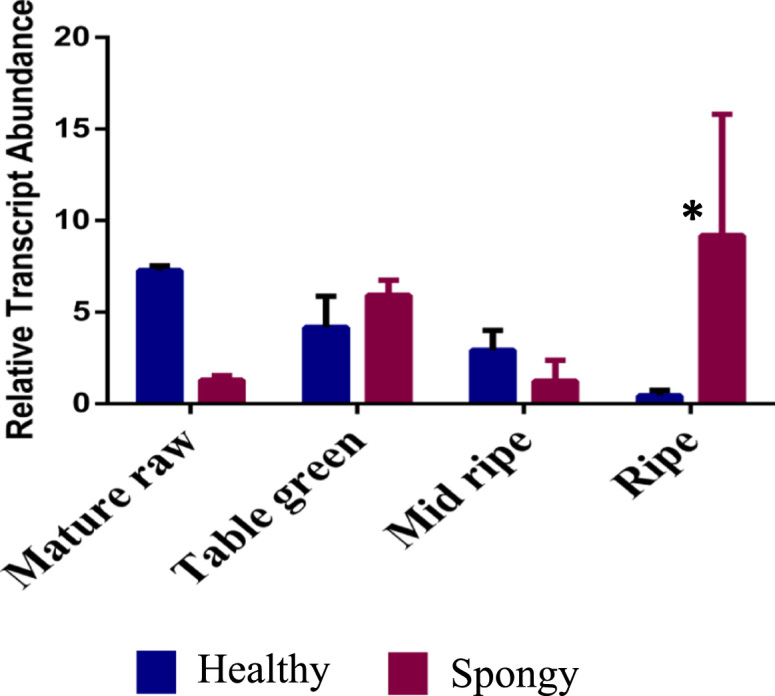
Relative transcript abundance – Relative transcript abundance of *Hydroperoxide Lyase* gene at ripening stages of Healthy and Spongy tissue.

Unsaturated fatty acids (Linoleic and α-Linolenic acid) are the precursors of lactone [3] and GLV biosynthesis [4]. In case of the spongy tissue, dominance of GLV and absence of lactones, along with increased concentration of Linoleic acid suggests the probable shifting of lactone biosynthesis pathway towards oxylipin biosynthesis pathways of GLV production. Hence, transcript abundance of 13-Hydroperoxide lyase (HPL) gene, involved in conversion of unsaturated fatty acid hydroperoxide to green leafy volatile [4] was also studied (Fig. 8). In case of the spongy tissue, 4 to 6-fold increase in gene expression of 13-HPL was observed as compared to the healthy fruits. A strong correlation (0.97) between transcript abundance and total GLV content at table green and mid ripe stages of spongy tissue suggests a probable involvement of oxylipin biosynthesis pathway of GLV production rather than lactone biosynthesis pathway at ripening stages of Alphonso mango with spongy tissue malady.

## 2 Experimental design, material and methods

### 2.1 Plant material

All the tissues of cv. Alphonso were collected from mango orchards of Agronomy department of the Dr. Balasaheb Sawant Konkan Agricultural University, Dapoli (N17°45′ E73°11′). To evaluate complete development of spongy tissue formation, four ripening stages of mango fruit pulp were collected [1]. Fruits of 0, 5, 10 and 15 DAH (Days After Harvest) (termed as mature raw, table green, mid ripe and ripe, respectively) were used for the present analysis. At each ripening stage mangoes were removed from hay boxes, and spongy affected mesocarp was separated and frozen in liquid nitrogen. Along with the spongy part non-spongy mesocarp around the spongy affected area was frozen separately and considered as spongy control. Completely healthy fruits i.e. free from the spongy tissue at the corresponding stage of ripening were also considered in this analysis. For statistical validation, fruits were collected from 5 individual trees which were considered as biological replicates.

### 2.2 Volatile extraction

Extraction of volatiles were done using 2 g mesocarp tissue from all the four ripening stages dichloromethane: acetone (80:20) as solvent system with appropriate concentration of nonyl acetate as an internal standard. Volatile extraction protocol was carried out as described previously [5].

### 2.3 Biochemical analysis of ripening related enzymes

Crude enzymes were used in enzyme assays which were extracted in HEPES-NaOH buffer (pH 7.4). Enzyme activity was calculated based on the amount of pNp released. Enzyme extraction and activity assay were performed as reported earlier [6].

### 2.4 Fatty acid extraction

Transesterification of fatty acids were carried out using methanolic HCl. One g of the mesocarp tissue was finely crushed in liquid nitrogen and added to the 5 ml methanol containing 3 M HCl, 25 µg butylated hydroxytoluene (BHT) as an antioxidant and appropriate amount of tridecanoic acid as an internal standard. FAME were extracted in n-Hexane and reconstituted in Chloroform. Transesterification and FAME extraction were carried out as reported earlier [3].

### 2.5 Gas chromatographic analysis

#### 2.5.1 Volatile identification and quantification

Gas chromatographic separation of volatiles was carried out on GsBP-5MS (General Separation Technologies, Newark, DE) capillary column (30 m × 0.32 mm i.d. × 0.25 μm film) with 1 ml min^−1^ flow of Helium as carrier gas. Oven temperatures were programmed from 40 °C for 5 min, raised to 180 °C at 5 °C/min followed by an increase till 280 °C at the rate of 20 °C/min and held at 280 °C for 5 min. General chromatographic and mass spectrometric conditions were retained as reported earlier [5]. Compounds were identified by matching generated spectra with NIST 2011 and Wiley 10th edition mass spectral libraries. Quantitative analysis was done using flame ionization detector maintaining the same chromatographic conditions. Absolute quantification was performed using known concentration of nonyl acetate (internal standard).

#### 2.5.2 Fatty acid analysis

Fatty acid separation was carried with SP^™^ 2560 (Supelco, Bellefonte, Pennsylvania, U.S.A.) column with 75 m long, 0.18 mm i.d. and 0.14 µm film thickness. Qualitative analysis was carried out on 7890B GC system Agilent Technologies coupled with Agilent 5977A MSD (Agilent technologies^®^, CA, U.S.A.) using 1 µl chloroform reconstituted FAMEs. Other gas chromatographic parameters were maintained as reported earlier [7]. Identified FAMEs were confirmed by spectral matching with NIST 2011 and Wiley 10^th^ edition mass spectral libraries. Compounds were validated by matching retention time and spectra of authentic standards procured from Sigma Aldrich (St. Louis, MO, USA). Quantification of identified compounds were done using GC-FID. Chromatographic conditions were similar for GC-MSD and GC-FID. Absolute quantification was done by normalizing concentrations of all the FAMEs with internal standard (tridecanoic acid methyl ester) [3].

### 2.6 Statistical analysis

For statistical validation of volatiles and fatty acids, at each stage of the ripening a minimum of two fruits of each of the five plants collected were used for independent extractions while each extract was analyzed twice on the GC. Fischer׳s LSD test (*p* ≤ 0.05) was carried out by ANOVA (StatView software, version 5.0 (SAS Institute Inc., Cary, NC, USA)) on healthy, spongy and spongy control tissue separately to compare the quantity of each compound and class within three datasets of four ripening stages. Principle component analysis for the whole data set of fatty acid content and volatile content was carried out using Systat^®^ statistical software (Version12, Richmond, CA, U.S.A.).

### 2.7 RNA isolation and cDNA synthesis

Total RNA isolation was carried out for all the tissues sampled for current study using RNeasy Plus mini kit (Quiagen, Hilden, Germany). RNA quality and integrity was checked using Bioanalyzer 2100 (Agilent Technologies, Santa Clara, USA). Two microgram of total RNA was used to carry out reverse transcription for synthesis of cDNA using High Capacity cDNA reverse transcription kit (Applied Biosystem, Carlsbad, CA, USA) [8].

### 2.8 Quantitative real-time PCR

Quantitative real-time PCR was performed using the Fast Start Universal SYBR Green master mix (Roche Inc. Indianapolis, Indiana, USA) and elongation factor 1α (EF1α) as an endogenous reference gene for which primers were reported earlier [9]. Hydroperoxide lyase gene was amplified using gene specific primers (MiHPL_F1 CGTCCTTGACATTCTGAAACGC and MiHPL_R1 CCTTCGCAGAGATGCTTGTTTC) covering amplicon size of 100 bp. Quantification of transcripts were done by ViiA™ 7 Real-Time PCR System (Applied Biosystems, California, USA) having thermal cycle program of initial denaturation at 95 °C for 10 min with subsequent 40 cycles of 95 °C for 3 s and 60 °C for 30 s followed by a melting curve analysis of transcript. Relative quantification (ΔΔCT method) and statistical analysis was carried out manually. Complete analysis was repeated with three biological replicates and three technical replicates were employed for each biological replicate.
