# Supplementary material for: Data on metabolic profiling of spongy tissue disorder in Mangifera indica cv. Alphonso
Source: Data Brief. 2018 Dec 4;22:145–57. doi: 10.1016/j.dib.2018.11.140 (PMC6299125; doi:10.1016/j.dib.2018.11.140)
Supplement: Supplementary file 1 — Supplementary material [file mmc1.docx]

All the authors declare that they have no conflict of interest.
